# Nutritional status of cancer outpatients using scored patient generated subjective global assessment in two cancer treatment centers, Nairobi, Kenya

**DOI:** 10.1186/s40795-017-0181-z

**Published:** 2017-08-10

**Authors:** Yvonne Opanga, Lydia Kaduka, Zipporah Bukania, Richard Mutisya, Ann Korir, Veronica Thuita, Moses Mwangi, Erastus Muniu, Charles Mbakaya

**Affiliations:** 1 School of Public Health, Moi University, P.O. Box 24405-00100, Nairobi, Kenya; 2 Centre for Public Health Research, Kenya Medical Research Institute, Nairobi, Kenya; 3 Centre for Clinical Research, Kenya Medical Research Institute, Nairobi, Kenya; 4 Department of Nutrition, Kenyatta National Hospital, Nairobi, Kenya; 5 Rongo University College, Migori, Kenya

**Keywords:** Cancer, Outpatients, Malnutrition, Subjective global assessment Kenya

## Abstract

**Background:**

Malnutrition is a universal problem in cancer patients renowned as an important factor for increased morbidity, decreased quality of life and high mortality. Early diagnosis of malnutrition risk through nutrition screening followed by comprehensive and timely interventions reduces mortality associated with malnutrition. The Scored Patient-Generated Subjective Global Assessment (PGSGA) method has been proved efficient in identifying cancer patients with nutrition challenges and guiding appropriate interventions. However this tool has not been adopted in management of cancer patients in Kenya. The aim of the study was to assess and describe nutrition status of cancer outpatients receiving treatment at Kenyatta National Hospital Hospital (KNH) and Texas Cancer Centre (TCC).

**Methods:**

The study adopted a hospital based descriptive cross sectional study. Cancer outpatients with confirmed stage 1–4 cancers, physically stable, aged 18 years and above and receiving cancer treatment were recruited and assessed using Scored PGSGA tool. Proportions, measures of central tendency and pearsons’ chi-square test were used in statistical analysis.

**Results:**

Among the 471 participants assessed, 71.8% were female and 28.2% male. Most participants had stage 2, 3 and 4 cancers at 27.2%, 27.2% and 24.3% respectively. Highest proportion of participants had breast (29.7%) and female genital cancers (22.9%). Sixty nine percent of participants were well nourished (SGA-A), 19.7% moderately malnourished (SGA-B) and 11.3% severely malnourished (SGA-C) and this difference was statistically significant. The mean PGSGA score was 6.76 (SD 5.17). Based on the score, 33.8% of participants required critical nutrition care, 34.8% symptoms management, 14.2% constant nutrition education and pharmacological intervention while 17.2% required routine assessments and reassurance. More (m;54.7%, f; 45.3%) males than females were severely malnourished(SGA-C) and this was statistically significant (*P* < 0.001).Prevalence of severe malnutrition was highest among participants with digestive organ cancers (49.1%) followed by those with lip cancer (17%) and the least prevalence reported in those with Karposi Sarcoma (0%). Most of stage 4 participants were moderately (37.5%) and severely (29.4%) malnourished.

**Conclusions:**

The Scored Patient-Generated Subjective Global Assessment is able to identify cancer patients both at risk of malnutrition and those severely malnourished. It also provides a guideline on the appropriate nutrition intervention hence an important tool in nutrition management of cancer patients.

## Background

Malnutrition is a universal problem in cancer patients renowned as an important factor for increased morbidity, decreased quality of life, decreased survival and high mortality [1]. In addition, malnutrition has been observed to negatively impact patients’ reaction to treatment, elevate treatment side effects, disrupt consecutive treatment regimens, increase hospital stay, weaken functionality and immunity of patient hence affecting survival rates of the patients [2] Malnutrition and weight loss are prevalent in 20–80% of cancer patients [3, 4]. It is characterized with depression, fatigue and malaise which also significantly impact patient well-being and is highly associated with significant healthcare costs [2]. Under nutrition often occurs as a result of an array of factors including reduced food intake, adverse effects from the anticancer treatment and altered metabolic processes due to the tumor [1]. Therefore, early recognition and detection of risk for malnutrition through nutrition screening followed by comprehensive nutrition assessment and timely interventions should be considered a valuable measure within the overall oncology strategy [5].

Scored Patient Generated Subjective Global assessment tool (PGSGA) [6] has been validated and accepted by the oncology nutrition dietetic group of American Dietetic Association as a standard tool for nutrition assessment of patients with cancer [3, 7]. It is a simple bedside method of assessing the risk of malnutrition and identifying those who would benefit from nutritional support hence considered to be the most appropriate tool for detecting malnutrition in cancer patients [8]. PGSGA relies majorly on weight history, changes in dietary intake of the patients, presence of gastrointestinal symptoms, functionality, and physical examination. The patients are then classified into three categories; Well nourished (SGA-A), Moderately undernourished or suspected malnutrition (SGA-B) and severely undernourished (SGA-C). The resulting total score guides the intervention plan [9]. Despite being identified as an ideal method of assessing nutrition status of cancer patients, the tool has not been utilized for the nutrition assessment of cancer patients in Kenya. The study therefore aimed to assess and describe the nutrition status of cancer outpatients receiving treatment at Kenyatta National Hospital and Texas Cancer Centre using the PGSGA tool.

## Methods

### Study design

A hospital based descriptive cross sectional study was adopted to assess and describe the nutritional status of cancer outpatients receiving treatment at Kenyatta National Hospital and Texas Cancer Centre.

### Study population

Cancer outpatients with established stage 1–4 cancers, aged 18 years and above who were physically stable with no active illness and body weakness were recruited while excluding the terminally ill patients and those who declined to consent.

### Sample size estimation and allocation

A sample of 512 participants was recruited for the study with a total of 312 and 200 participants from KNH and TCC respectively. The Fishers et al. formula [10] was used to calculate the required minimum sample size of 385 with 95% confidence interval, error margin of 0.05 based on an assumed prevalence of malnutrition of 50% among cancer patients in Kenya. The required *n* = 385 and assuming 10% non response rate, the study aimed at recruiting a minimal sample of 424 patients. The calculated sample size was distributed between the two facilities using square root allocation method to ensure that the two facilities were equally represented based on the number of patients received per facility per year. Participants were recruited on the days they visited the hospital for treatment. The nurses list of attendance for patients was used to select the patients using systematic random sampling method where every third patient was recruited until the desired sample size was achieved. Seven qualified nutritionists with past experience in nutrition assessments and health facility based data were recruited as research assistants from a pool of existing institutional personnel database. In addition, four hospital personnel (one nurse and one health records officer per facility) were recruited to assist in patient record retrieval, patient identification and coordination. The team was trained on how to administer the PGSGA tool, assess the patients and ethical considerations during data collection.

### Methodology

The Standard PGSGA tool developed and validated for use in ambulatory oncology settings was used for data collection. Sections on weight history, changes in dietary intake, presence of nutrition impacting symptoms and functionality were assessed by trained nutritionists while physical examination was carried out by experienced nurses at both facilities. Patients current weight and height were measured using SECA Scales and stadiometers respectively. Information on dietary intake and nutrition symptoms was reported by participants while information on weight history, use of corticosteroids, type of cancer stage and any other illness was retrieved from participants medical records. Each participant was classified as well nourished (SGA-A), moderately malnourished (SGA-B) and severely malnourished (SGA-C). The total PGSGA score was determined by summing up the scores for all the sections (weight history, dietary intake, nutrition impact symptoms). The scores were further classified according to corresponding nutrition intervention such that a score of 0–1 inicated regular reassessment and reassurance during treatment; 2–3- Patient and family education with pharmacological intervention; 4–8 intervention on symptom management by dietitian and a score of >9; indicates a critical need for nutrient support options [9]. Table 1 shows the PGSGA Global assessment categories used in patient classification.

**Table 1:** PGSGA Global assessment categories

Category	SGA-A	SGA- B	SGA-C
	Well nourished	Moderate/suspected malnutrition	Severely malnourished
Weight Change	No weight loss or recent non fluid weight gain	≤ 5% loss in 1 month (≤10% in 6 months) or progressive weight loss	> 5% loss in 1 month (>10% in 6 months) or progressive weight loss
Nutrient Intake	No deficit **or** Significant recent improvement	Definite decrease in intake	Severe deficit in intake
Nutrient Impact Symptoms(NIS)	None **or** significant recent improvement allowing adequate intake	Presence of NIS	Presence of NIS
Functioning	No deficit **or** Significant recent improvement	Moderate functional deficit **or** Recent deterioration	Severe functional deficit **or** Recent significant deterioration
Physical Exam	No deficit **or** chronic deficit but with recent clinical improvement	Evidence of mild to moderate loss of muscle mass	Obvious signs of malnutrition (severe loss muscle fat, oedema)

Source [9]

Data on clinical characteristics of the participants including the cancer staging and type were extracted from participants’ medical records. In Kenya, cancer staging is carried out using the Tumor Node Metastasis (TNM)Staging system [11]. Cancer classification is established by the International Classification of Diseases for Oncology, third edition (ICD-O-3) which classifies cancer based on either the tissue from which the cancer originates (histology types), primary site or the location in the body where cancer first developed [12].

### Statistical analysis

Data were entered in MS Access database, cleaned and exported to SPSS version 20.0 software for data analysis. Exploratory data analysis techniques were used at the initial stage of analysis to uncover the data structure and identify outliers or unusually entered values. Proportions were used to summarize categorical variables and measures of central tendency for continuous variables. Pearson’s Chi-square test or fisher exact test was used to assess the relationship between dependent and categorical variables. The threshold for statistical significance was set at α = 0.05 (two-sided).

## Results

A total of 41 patients whose weight history details were missing were excluded from analysis hence the study presents results of 471 participants who had complete data on weight history.

Among the 471 participants assessed, 71.8% were female and 28.2% male. The mean (SD) age of participants was 52 years (13.7). Most participants had stage 2, 3 and 4 cancers at 27.2%, 27.2% and 24.3% respectively. Highest proportion of participants had breast (29.7%) and female genital cancers (22.9%) and lip, oral cavity and pharynx cancers (18.5%) as shown in Table 2.

**Table 2:** Participant demographic and clinical characteristics

Variable		Number	Percent
Sex	Female	338	71.8
	Male	133	28.2
Age	Mean(SD) 51.6(13.7)		
Stage of cancer	Stage 0	3	.7
	Stage 1	69	15.1
	Stage 2	124	27.2
	Stage 3	124	27.2
	Stage 4	111	24.3
	Don’t know	25	5.5
Type of cancer	Lip, oral cavity and pharynx	87	18.5
	Respiratory organs	7	1.5
	Bone, cartilage, melanoma	16	3.4
	Breast	140	29.7
	Female genital	108	22.9
	Haematopoietic	8	1.7
	Kaposi sarcoma	6	1.3

Results showed that more than half (69%) of the participants were well nourished (SGA-A), 19.7% moderately malnourished or had suspected malnutrition (SGA-B) and 11.3% severely malnourished (SGA-C). A mean (SD) PGSGA score of 6.76 (5.17) was reported. When classified based on the type of nutrition intervention, the study indicated that 17.2% of the participants required minimal nutrition intervention with routine assessments and reassurance, 14.2% required constant nutrition education for both the patient and the caregivers, pharmacological intervention based on biochemical analysis, 34.8% required interventions on symptoms management while 33.8% require critical nutrition care as shown in Table 3.

**Table 3:** Nutrition status of the study participants by Patient Scored Subjective Global assessment

Variable	Description	Number	Percent
Nutrition status	Well-nourished (SGA-A)	325	69.0
	Moderate or suspected Malnutrition(SGA-B)	93	19.7
	Severely Malnourished(SGA-C)	53	11.3
PGSGA score	Mean(SD)	6.76(5.17)	
PGSGA score classification	Reassurance and follow up	81	17.2
	Nutrition Education	67	14.2
	Symptom management/Nutrition counseling	164	34.8
	Critical Nutrition Care	159	33.8

The study results showed a statistically significant difference in the mean PGSGA scores for each of the PGSGA classification of nutrition status (*p* < 0.001) with the highest proportion (86.8%) of severely malnourished participants having the highest scores. More females (79.4%) than males (20.6%) were well nourished SGA-A, however, the reverse observed with severe malnutrition SGA-C(m;54.7%; f; 45.3%) indicating a statistically significant difference in nutrition status by sex types (*P* < 0.001). The study also showed a statistically significant difference (*p* < 0.001) in nutrition status and cancer staging with highest proportions (31.5%) of participants with stage 2 cancers being well nourished and most stage 4 cancer participants presenting with moderate and severe malnutrition (SGA-B; 37.5%; SGA-C 29.4%). When ranked based on the cancer types, findings indicated a significant association with the level of malnutrition (*p* < 0.001). For instance, results revealed that the prevalence of severe malnutrition was highest among participants with digestive organ cancers (49.1%) followed by those with lip cancer (17%) and the least prevalence reported in those with Karposi Sarcoma (0%). Most participants with breast and female genitalia cancers were well nourished SGA-A at 37.2 and 24.2% respectively as shown in Table 4.

**Table 4:** Nutrition status of participants by demographic and clinical characteristics

Variables	Description	SGA-A n(%)	SGA-B n(%)	SGA-C n(%)	PVALUE
PGSGA Score Classification	Reassurance	78(24)	3(3.2)	0(0)	<0.001
	Nutrition Education	57(17.5)	10(10.8)	0(0)	
	Symptom Management	120(36.9)	37(39.8)	7(13.2)	
	Critical Care	70(21.5)	43(46.2)	46(86.8)	
Sex	Male	67(20.6)	37(39.8)	29(54.7)	<0.001
	Female	258(79.4)	56(60.2)	24(45.3)	
Cancer Staging	stage 0	3(0.9)	0(0)	0(0)	0.002
	stage 1	46(14.5)	13(14.8)	10(19.6)	
	stage 2	100(31.5)	16(18.2)	8(15.7)	
	stage 3	93(29.3)	17(19.3)	14(27.5)	
	Stage 4	63(19.9)	33(37.5)	15(29.4)	
Typeof cancer	Lip, oral cavity and pharynx	55(16.9)	23(24.7)	9(17)	<0.001
	Digestive organs	48(14.8)	25(26.9)	26(49.1)	
	Respiratory organs	2(0.6)	3(3.2)	2(3.8)	
	Bone, cartilage, melanoma	8(2.5)	5(5.4)	3(5.7)	
	Breast	121(37.2)	15(16.1)	4(7.5)	
	Female genital	81(24.9)	20(21.5)	7(13.2)	
	Haematopoietic	4(1,2)	2(2.2)	2(3.8)	
	Kaposi sarcoma	6(1.8)	0(0)	0(0)	

## Discussions

This study assessed the nutritional status of cancer outpatients receiving treatment at two cancer treatment centers in Nairobi Kenya using scored PGSGA tool. Results revealed that 31% of the patients were undernourished (11.3% severely malnourished and 19.7% moderately malnourished). The findings show a statistically significant difference in nutrition status with more males (54.7%) than females (45.3%) reported to be severely malnourished (*p* < 0.001). There was a statistically significant (*p* < 0.001) difference in nutrition status among patients with different cancer stages with more stage 4 patients being moderately and severely malnourished than patients in other stages. A mean(SD) PGSGA Score of 6.79(5.17) was established and this indicated that nearly all patients require symptom management as indicated in the Ottery guidelines [9]. When categorized based on the type of intervention the second highest proportion (33.8%) of participants required critical nutrition care (Score > 9).

Results revealed that 31% of the patients were undernourished (11.3% severely malnourished and 19.7% moderately malnourished). Malnutrition has been known to occur mostly as a comorbidity among cancer patients with an estimated prevalence of 20–80% [2, 3, 13]. Combined effects of the cancer and treatment option often predispose cancer participants to nutrient depletion and inadequate food intake resulting in poor nutritional profiles [14–16].

Results from our study were consistent with those from an Australian study which showed that 17% of cancer outpatients were severely malnourished based on an assessment carried out using subjective global assessment [7]. Although showing a slightly lower prevalence, our results were also consistent with findings from a study carried out among elderly cancer patients which showed that 14.6% of participants were severely malnourished [17]. PGSGA is used as an assessment tool that better identifies established malnutrition than nutritional risk [18]. In Kenya, malnutrition remains a challenge among cancer outpatients in Kenya because most of the nutrition interventions are carried out among cancer inpatients [19]. A study carried out among cancer patients established that only 18% of cancer outpatients reported to have received nutrition services [20]. Majority of these patients have limited information on appropriate nutrition practices during cancer treatment and rely on myths and misconceptions that later influence dietary intake resulting to poor nutrition status. As much as Kenya is faced with these challenges compared to the developed world, this study provides opportunities that can be tapped into to improve nutritional outcomes for cancer patients. For instance, there is need to scale up nutrition interventions for cancer outpatients, develop appropriate guidelines for managing side effects of treatment and promoting better practices among cancer patients especially while they are at home. There is also need for appropriate education and counselling for caregivers of these patients to prevent malnutrition.

The findings show a significant difference in nutrition status with more males (54.7%) than females (45.3%) reported to be severely malnourished (*p* < 0.001). In Kenya, among male cancer patients, majority suffer from prostate, oesophageal and colorectal cancer compared to women who present with breast and cervical cancer [21]. Studies have revealed that digestive organ cancers; Oesophageal, Colorectal and Stomach cancer present a higher risk of malnutrition compared to cancers related to reproductive system [22] hence men in Kenya are more likely to suffer malnutrition compared to women on the basis of cancer type. In addition, men are more likely to experience severe effects from cancer compared to women because of their poor health seeking behaviors; they often seek for medical attention at a later stage when the damage is already done [23]. Moreover, the Kenya Demographic Health Survey indicates that one third (33%) of women in Kenya are overweight and obese with only 9% undernourished [24] hence lower cases of severe malnutrition in women compared to men. Therefore, there is need for nutritionists managing cancer patients to continuously offer nutrition support, nutrition education, counselling and follow up of male cancer patients. There is also need to carry out training, provide information to empower male cancer patients on the role of nutrition therapy in improvement of treatment outcomes.

Our findings show that stage 4 patients were significantly (*p* < 0.001) moderately and severely malnourished compared to patients in the other stages. Our findings concur with results from a study carried in Korea among hospitalized cancer patients with advanced stages (60.5%) who had a higher prevalence of malnutrition than other patients (*p* < 0.0001). Similarly, patients with digestive organ and lip cancers had higher levels of under nutrition compared to those with breast and cancers that affect female reproductive organs. Studies have shown that participants with lip/oral cancers report highest levels of malnutrition [16] hence need for timely nutrition intervention among these patients. Most of these patients experience dysphagia hence consume less food especially in cases where enteral or parenteral modes of feeding have not been initiated. In a similar study among 498 participants with advanced GI cancers in Beijing, results identified that 54% required improved nutrition support with PGSGA score of ≥9 [8]. Evidence has shown that the prevalence of malnutrition depends on the type, location, stage of tumor, and type of treatment used [7, 25]. Our findings concur with a study carried out to determine factors influencing nutrition status among cancer patients which indicated that malnutrition is related to type and site of origin of tumor and in early stages of disease is more pronounced in patients with oesophagus and stomach cancer [26]. In addition the same study showed that malnutrition gets more severe as the disease progresses to advanced stages except for breast and cervical cancer [26]. Another study among women with female genital tumors showed no significant difference in nutrition status by PGSGA according to different cancer stages [27]. In settings with limited dieticians as Kenya, provision of systems and guidelines for malnutrition based on the cancer type will be ideal. There is need to promote early diagnosis of malnutrition and create awareness on management of cancer and treatment side effects that lead to undernutrition among cancer patients for effective outcomes.

A PGSGA Score of 6.79 (5.17) in the findings is an indication that nearly all patients require symptom management as indicated in the Ottery guidelines [9]. When categorized based on the type of intervention the second highest proportion (33.8%) of participants require critical nutrition care (Score > 9). A similar study carried out in India among cancer participants showed that 35.7% of participants had PG-SGA score between 4 and 8 hence require intervention by dietitian; 20% had score > 9 hence recommended critical nutritional intervention [3]. The scored PGSGA tool is unique such that it helps identify malnourished hospital participants as well as give guidelines for triaging patients for nutrition intervention. Additionally, the score helps identify impact of symptoms on nutrition status of the participants which in turn impacts on treatment outcomes and prognosis therefore highly recommended for use in decision making on appropriate nutrition care processes for cancer patients [3].

PGSGA tool being subjective, relies heavily on information reported by the patients especially on dietary history and changes in the physiological state of the patients in the past 2 weeks and 1 month. One of the study limitations was the recall bias where some patients could not recall changes in their dietary practices in the past 1 month or past 2 weeks, and in a few circumstances the weight history. In such scenarios, research assistants probed for more and sought clarification from caregivers where necessary. In addition, information on patient’s weight history was extracted from medical records. In cases where the information was missing, reported weight by the patients was used.

## Conclusions

Nutrition assessment is necessary for timely nutrition interventions for cancer patients. It also helps to prevent further or pending malnutrition and weight loss during treatment and ultimately improve the quality of life of persons with cancer. As indicated by the results, the PGSGA tool is able to identify cancer patients both at risk of malnutrition and those severely malnourished and also provides a guideline on the type of nutrition intervention. There is therefore a need for adoption of the tool in nutrition management of cancer outpatients in Kenya.

## Abbreviations

CPHR: Centre for Public Health Research
GI: Gastrointestinal
KEMRI: Kenya Medical Research Institute
KNH: Kenyatta National Hospital
PGSGA: Patient Generated Subjective Global Assessment
SD: Standard deviation
SERU: Scientific and Ethics Review Unit
SGA-A: Subjective Global Assessment Anabolic
SGA-B: Subjective Global Assessment Between Anabolic and Catabolic
SGA-C: Subjective Global Assessment Catabolic
TCC: Texas Cancer Centre
